# Reducing the carbon footprint for a 30-bed haemodialysis unit by changing the delivery of acid concentrate supplied by individual 5 L containers to a central delivery system

**DOI:** 10.1007/s40620-024-02073-9

**Published:** 2024-09-18

**Authors:** Gareth Murcutt, Rosie Hillson, Cate Goodlad, Andrew Davenport

**Affiliations:** 1 UCL Department of Renal Medicine, Royal Free Hospital London, Pond Street, London, NW3 2QG UK; 2 Centre for Sustainable Healthcare, 8 King Edward Street, Oxford, OX1 4HL UK; 3 Department of Medicine, University College London, Gower Street, London, WC1E 6BT UK

**Keywords:** Greenhouse gases, Haemodialysis, Carbon foot print, Acid concentrate, Dialysate

## Abstract

**Background:**

Haemodialysis treatments generate greenhouse gas (GHG) emissions mainly as a result of the equipment, consumables and pharmaceuticals required. An internal audit demonstrated a 33% wastage of acid concentrate when using individual 5.0 L containers at a 1:44 dilution ratio. We therefore investigated whether changing the delivery system for acid concentrate would reduce wastage and any associated greenhouse gas emissions.

**Methods:**

We calculated the difference for a 30-bed dialysis unit between receiving acid concentrate in single-use 5.0 L plastic containers versus bulk delivery for a central acid delivery system connected to the dialysis machines. Estimates of carbon dioxide equivalent (CO_2_e) emissions were made using the United Kingdom government database and other sources.

**Results:**

A 30-station dialysis unit functioning at maximum capacity (3 shifts and 6 days/week), switching to bulk delivery and central acid delivery could realise an approximate total reduction of 33,841 kgCO_2_e/year; in reduced product wastage, saving 6192 kgCO_2_e, 5205 kgCO_2_e from fewer deliveries, and 22,444 kgCO_2_e saving from a reduction in packaging and waste generated, which equates approximately to a one tonne reduction in CO_2_e emissions per dialysis station/year.

**Conclusions:**

Switching from delivering acid concentrate in individual 5.0 L containers to a central acid delivery system can result in substantial reductions in CO_2_e emissions within a dialysis clinic. The emission savings from reducing the single-use plastic packaging greatly outweigh any gains from eliminating wastage of acid concentrate. Dialysis companies and clinicians should consider reviewing the design of current and future dialysis facilities and policies to determine whether reductions in CO_2_e emissions can be made.

**Graphical Abstract:**

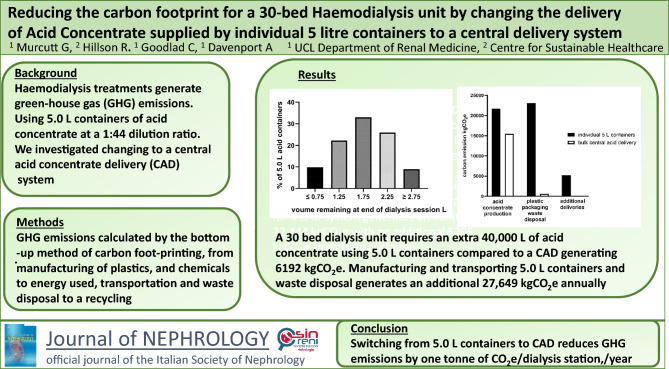

**Supplementary Information:**

The online version contains supplementary material available at 10.1007/s40620-024-02073-9.

## Background

To reduce the impact of anthropogenic climate change [1], the United Kingdom (UK) National Health Service (NHS) adopted the “Delivering a ‘net zero’ NHS Service” plan in 2022 [2], which targets net zero by 2040 for the emissions the NHS directly controls and net zero by 2045 for the emissions it could influence [3]. Albeit a well-established treatment for patients with chronic kidney disease, haemodialysis is widely recognised as a carbon intensive treatment, with estimates that treating a patient for one year generates between 7.1–10.2 tonnes of carbon dioxide equivalent (CO_2_e) emissions [4, 5]. This is some 18 times more than that estimated of 0.4 tonnes CO_2_e per NHS patient per year [6]. Therefore, just as dialysis is considered a high-cost low volume treatment in financial terms, it is also a carbon intensive treatment.

**Fig. 1:**
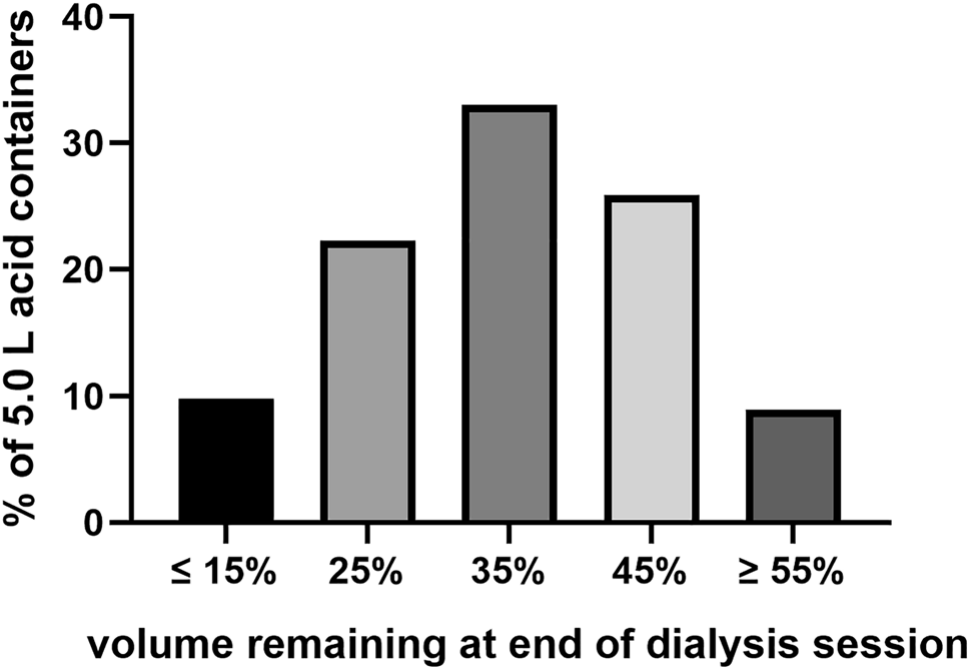
Internal Department of Renal Medicine audit data from the measurement of acid concentrate remaining in 106 5.0 L acid concentrate containers at the completion of a haemodialysis session

To achieve substantial reductions in greenhouse gas emissions, it is important to understand the source of greenhouse gas emissions associated with dialysis. For kidney care services, the majority of emissions (73%) are attributed to the procurement of medical equipment, consumables and pharmaceuticals [4]. Acid concentrate is an essential consumable used in all haemodialysis treatments and is mainly supplied within the UK in 5.0 L plastic containers for dilution at a 1:44 ratio, 6.0 L containers for dilution at a 1:34 ratio or bulk Central Acid Delivery at either of the above dilution ratios. Our university hospital kidney dialysis service currently uses a single-use 5.0 L plastic container of 1:44 dilution acid concentrate for each dialysis treatment. In 2021 the Dialysis Technical Services team conducted an internal audit of the volume of concentrate remaining in the plastic container at the end of treatment. Across four dialysis units, on average one third of every 5.0 L container was being discarded at the end of treatment (Fig. 1).

In 2011, the Bradford Renal Unit reported on changing from using individual 6.0 L plastic containers to a Central Acid Delivery system [7]. One of the reported benefits was an annual reduction in greenhouse gas emissions estimated at 8.3 tonnes CO_2_e by reducing the amount of unused acid concentrate and a further annual 7.7 tonnes CO_2_e from decreasing waste disposal.

In view of the large amounts of acid concentrate wasted in our dialysis centres annually we initiated a project to investigate the effects of installing central acid delivery systems. The aim of this study was to estimate the difference in the carbon footprint of acid concentrate supplied in reusable bulk containers compared to our current practice of using individual single-use 5.0 L plastic containers within a 30-station kidney dialysis unit.

## Methods

Carbon foot-printing is a method of estimating the total greenhouse gas emissions associated with the production, use and disposal of a particular product or service. This study utilises a process based on the Bottom-Up method of carbon foot-printing [8]. In this approach, each part of the process, from the manufacturing of plastics and other chemical ingredients to energy consumed, is converted into a common currency, that of CO_2_e, which are then summed to provide the total greenhouse gas emissions [9].

In order to convert the available information into kgCO_2_e, we used a range of data sources including direct measurements, data provided by the original equipment manufacturer, the UK government Department of Environment, Food and Rural Affairs (DEFRA) and Department for Business, Energy and Industrial Strategy (BEIS) database of CO_2_e conversion factors, and expert opinion [10]. Where assumptions have been made, we have taken a conservative view with respect to any gains claimed.

Our acid concentrate is manufactured in Huthwaite, Nottinghamshire, UK which is assumed to be a standard distance of 200 km from our kidney care units and transported by road in typically 7.5–17.5 tonne diesel-fuelled commercial vehicles. The detailed methodology used to determine the carbon footprint data is included within the Supplementary Materials section.

Currently, acid concentrate is delivered in single-use 5 L high-density polyethylene containers, whereas for a central acid delivery system the concentrate is shipped in reusable 1000 L bulk containers. The only single use requirement for the bulk acid concentrate delivery is a 2.275 kg linear low-density polyethylene liner used within each bulk container (information provided by original equipment manufacturer). Our project will provide two different acid concentrates delivered via central acid delivery available at each dialysis station. The acid concentrate is pumped from the bulk containers into one of two 3000 L storage tanks from where it is again pumped around the kidney dialysis unit, with the twin pipework following the route of the ultrapure water ring main. An outlet for each concentrate is provided at all dialysis stations.

Data are presented as mean estimated values (Prism 10.2 Graph Pad, San Diego, USA). This paper is intended to enhance the knowledge of clinicians, rather than presenting a meticulous Life Cycle Analysis of the entire system, however the carbon footprints in the Supplementary Material were completed using the principles of ISO14040 [11].

### Ethics

This study did not require individual patient consent and obtained UK national health care ethical approval under the guidelines for service development (https://www.hra.nhs.uk).

## Results

The results are presented to demonstrate the potential greenhouse gas emission savings for a 30-bed dialysis centre operating three shifts, 6 days a week, totalling some 28,080 annual treatments. They are extrapolated from the calculations used to determine the emissions generated in producing 10,000 L batches of acid concentrate (see Supplementary material) and the 33% wastage associated (Fig. 1) with our current practice.

The 30-station dialysis unit described previously would require 140,400 L per annum of acid concentrate delivered in 5.0 L containers, which to fit our scale we have taken as 140,000 L. Eliminating the 33% wastage by utilising a central acid delivery system would reduce this demand to 93,338 L, however taking a conservative estimate, we have used the higher volume of 100,000 L of acid concentrate to be used annually.

**Fig. 2:**
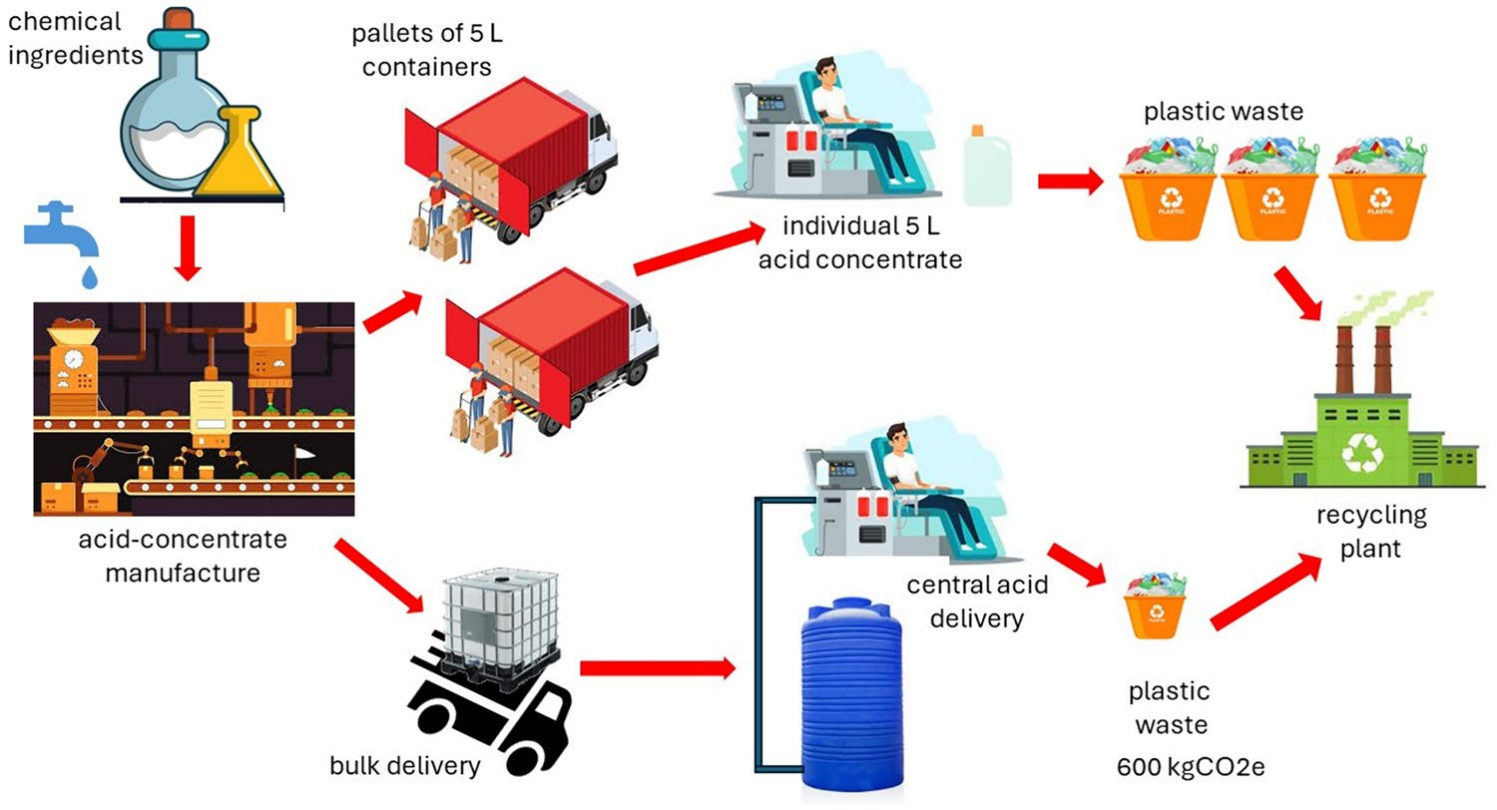
A 30-station dialysis unit functioning at maximum capacity (3 shifts and 6 days/week), then switching to bulk delivery and CAD could realise an approximate total reduction of 33,841 kgCO_2_e per year; as less acid concentrate is wasted, so reducing the amount required to be manufactured saving 6192 kgCO_2_e, and fewer deliveries so saving 5205 kgCO_2_e, and a 22,444 kgCO_2_e saving due to a reduction in packaging and waste generated

We have calculated the greenhouse gas emission savings associated with the non-production and delivery of these 40,000 L. In addition to the annual 6192 kgCO_2_e savings in manufacturing and production, there are also much lower greenhouse gas emissions due to the reduction in the requirement for single-use plastic packaging and waste disposal when using a central acid delivery system of 22,444 kgCO_2_e, along with savings from fewer deliveries, with an estimated total annual CO_2_e saving of 33,841 kgCO_2_e for a 30-bed dialysis unit (Table 1, Fig. 2).

**Table 1:** Summary of greenhouse gas savings for a 30-bed dialysis unit utilising a central acid delivery (CAD) system as opposed to using individual 5.0 L containers

Delivery method	Volume required, L	10,000 L batches required	Acid concentrate production kgCO_2_e	Plastic packaging/disposal kgCO_2_e	Additional deliveries (40,000 L)	Total kgCO_2_e
5.0 L containers	140,000	14	21,672	23,044	5205	49,921
CAD	100,000	10	15,480	600	0	16,080
Difference/saving	40,000	4	6192	22,444	5205	33,841

## Discussion

Although we accept that the science of calculating greenhouse gas emissions is evolving, and that the accuracy of calculating kgCO_2_e is in its infancy, the calculated emission saving of 33,841 kgCO_2_e is hugely significant. For a 30-bed dialysis unit, the 30 plus tonnes of CO_2_e/year emission savings would be equivalent to driving the average car 85,500 miles (136,800 km) [10], more than three times around the equator. Even with this inexact science, our calculations can be used to generate a useful metric in that installing central acid delivery will save in the region of 1000 kgCO_2_e per dialysis station per year. This reduction in the amount of acid-concentrate required would lead to an annual financial saving of £18,400 (€ 21,500) calculated at current UK prices [112]. Many factors influence the cost of installing a central acid delivery system but within this project we expect the savings in acid concentrate to pay for the installation over a 3-to-4-year period.

We expected that the majority of greenhouse gas savings would come from not manufacturing and delivering the additional 40,000 L of acid concentrate required when using individual 5.0 L plastic containers. The fact that the removal of the high-density polyethylene plastic containers alone generated 22,444 kgCO_2_e of savings came as a surprise. The weight of each 5.0 L high-density polyethylene container is 250 ± 5 g (see Supplementary materials) making the total weight of plastic required to deliver 140,000 L of acid concentrate equal to 7000 kg. In comparison the one hundred 1000 L linear low-density polyethylene liners required to deliver 100,000 L of concentrate have a total weight of 227.5 kg. This difference in the amount of plastic required, and subsequent waste disposal, accounts for the vast majority of the greenhouse gas savings associated with moving to a central acid delivery system. Thus, switching to a central acid delivery system reduces not only the amount of single-use plastic required to be manufactured, transported, and then disposed of, but also reduces the volume of acid concentrate required by the dialysis unit.

Though outside of the scope of this study, there will be other further reductions in greenhouse gas emissions by using central acid delivery. The transport emissions required to deliver 28,000 empty 5.0 L containers to the manufacturing site will undoubtedly be significantly more than when delivering 100 plastic liners. As well as this, the additional 40,000 L required to be manufactured if using 5.0 L containers will need additional manufacture and delivery of chemical consituents to the factory.

Overall, as well as the reduction in carbon emissions, switching to a central acid delivery system produces less waste to be disposed of, along with reduced staff manual handling and time saved setting up and clearing away after the dialysis session. Using a central acid delivery system and the subsequent saving of one third of each 5.0 L cannister will also, over time, reduce the financial cost of dialysis. The reduction in greenhouse gas would have been even greater if we had been supplied with 6.0 L plastic containers for dilution at a 1:34 ratio, rather than 5.0 L containers for dilution at a 1:34 ratio, in terms of water requirements for production of acid concentrate, additional size and weight and of containers for transportation and waste disposal.

We accept that the advantage of choosing individual 5.0 L acid concentrates allows individualisation of the dialysis prescription, but no dialysis centre has the storage capacity to offer patients every conceivable variation in calcium, potassium, magnesium, and acetate concentrations. As with most UK dialysis centres, we currently offer several different acid concentrates, and moving to a central acid delivery system would lead to a reduction in the number of available concentrates. However, most of our patients used one of two concentrates and some 5 L containers will continue to be used for patients requiring an individual prescription.

Some countries, such as Japan, have favoured a single-batch dialysate delivery system, delivering a fixed electrolyte and bicarbonate dialysate [13]. Although this system would potentially further reduce greenhouse gas emissions, this does not allow for any individualisation of dialysate prescriptions [14]. Thus, compared to a single dialysate delivery system, a central acid delivery system does allow flexibility in the final dialysate composition via a choice of concentrate and adjustable dialysis machine settings.

Other systems such as Granumix™ (Fresenius Medical Company, Bad Homburg, Germany) are available, whereby a dry acid concentrate powder is delivered, then diluted, and mixed within the dialysis unit, designed for use with a central acid delivery system. As such, this would potentially additionally reduce the transport weight of the water and thus greenhouse gas emissions, but the system is not currently marketed within the UK. Another alternative has been to supply 3.5 or 5.0 L pre-mixed acid concentrate in soft polyvinyl chloride plastic bags (SoftPac™, Baxter Health Care Corporation, Illinois, USA), but this approach is limited to a number of specific dialysis machines. Although lighter than the equivalent high-density polyethylene containers, these are still single-use and thus any emissions savings are likely to be small in comparison to a central acid delivery system.

Haemodialysis is a well-established treatment for patients with kidney failure world-wide, however treatments require large volumes of treated water, mixed with acid concentrate and bicarbonate. Although many authors have concentrated on reducing or recycling the amount of water used for dialysis treatments [15–17], this study demonstrates that the main environmental impact is caused by the amount of single use plastic required. Rizan and colleagues demonstrated the dramatic increase in greenhouse gas emissions associated with the increase in single use, rather than a circular system of recycling and re-using medical plastic waste [18]. Our study supports their conclusions. The environmental consequences of delivering the acid concentrate in single-use disposable plastic containers, along with the wastage of the actual product, is viewed as completely unsustainable.

## Conclusions

Not all dialysis units will be suitable for central acid delivery systems, however in this era of awareness of the deleterious effects of greenhouse gas emissions, dialysis companies and clinicians should review policies and standards of care, especially when designing new dialysis centres, to determine whether changes can lead to a reduction in CO_2_e emissions. Advances in water purification systems and dialysis machine technology can reduce the amount of water and dialysate required per session, but moving from individual single-use acid concentrate plastic containers to a bulk delivery of concentrates with a central delivery system will have an even greater impact on reducing greenhouse gases and should be considered when designing dialysis centres. As 2023 was widely judged to have been the warmest year so far on record, then potentially saving some 1000 kgCO_2_e per dialysis station annually should be considered by dialysis providers, as the kidney care community endeavours to reduce greenhouse gas emissions.

### Learning points

**Table:** 

**What was known**
Haemodialysis treatments are recognised to have an impact on the environment by generating greenhouse gases and increasing carbon emissions
Acid concentrate is an essential consumable used in all haemodialysis treatments and can be supplied in a variety of formats including 5.0 L plastic containers for dilution at a 1:44 ratio, 6.0 L containers for dilution at a 1:34 ratio and bulk Central Acid Delivery.
Supplying individual 5.0 or 6.0 L containers requires greater manufacture of plastics, transport and waste disposal.
**What this study adds**
We have demonstrated that moving from individual 5.0 L acid concentrate containers to a central acid delivery system significantly reduces the generation of greenhouse gases.
For a 30-bed dialysis unit there is an approximate reduction of some 30,000 kg of carbon emissions.
The bulk of the emissions savings came, not from reducing the wastage of the current system, but by the removal of single-use plastic containers from the supply chain.
**Potential impact**
Installing a central acid delivery system is estimated to save in the region of 1000 kgCO_2_e per dialysis station per year.
Designing dialysis units to have a central acid delivery system significantly reduces the environmental impact of haemodialysis treatments.

## Supplementary Information

Below is the link to the electronic supplementary material.

Supplementary file1 (DOCX 28 KB)

## Data Availability

Data available on reasonable request to authors.
