# Supplementary material for: Reducing the carbon footprint for a 30-bed haemodialysis unit by changing the delivery of acid concentrate supplied by individual 5 L containers to a central delivery system
Source: J Nephrol. 2024 Sep 18;37(7):1949–55. doi: 10.1007/s40620-024-02073-9 (PMC11519164; doi:10.1007/s40620-024-02073-9)
Supplement: Supplementary file 1 — Supplementary file1 (DOCX 28 KB) [file 40620_2024_2073_MOESM1_ESM.docx]

**Reducing the carbon footprint for a 30-bedded Haemodialysis unit by changing the delivery of Acid Concentrate supplied by individual 5 litre containers to a central delivery system**

^1^Gareth Murcutt, ^2^Rosie Hillson, ^1^Cate Goodlad, ^3^Andrew Davenport

^1^UCL Department of Renal Medicine, Royal Free Hospital London, London NW3 2QG, UK

^2^Centre for Sustainable Healthcare, 8 King Edward Street, Oxford OX1 4HL, UK

^3^Department of Medicine, University College London, Gower Street, London WC1E 6BT UK

**Supplementary Material:**

Words

Body 1456

Tables 4

References 6

**Introduction**

This section describes the detailed methodology of obtaining the carbon footprints of the products and systems quoted in the main study. Information from the Original Equipment Manufacturer (OEM) was combined predominantly with the UK government Department of Environment, Food and Rural Affairs (DEFRA) and Department for Business, Energy and Industrial Strategy (BEIS) database of kgCO_2_e conversion factors and included some internet searches where required. A 10,000 litre batch of acid concentrate was used for the calculations, the results of which were then extrapolated into the volumes required for the 30-bedded unit described in the main study.

**Assumptions and Reasoning:**

A typical dialysis acid concentrate contains purified water, different chloride salts (predominantly sodium chloride), as well as quantities of acetic acid and glucose. In the absence of publicly available data for the production of highly purified chemicals and using information provided by the OEM (Fresenius Medical Care UK Ltd) (Table 1) this study assumes that each 10,000-litre batch of 1:44 dilution acid concentrate is composed of:

8,836 litres of ultrapure water

2,924 kg of purified sodium chloride

512 kg of Glucose

98 litres/103kg of Acetic Acid

Sodium Chloride:
The carbon emissions factor associated with sodium chloride (table salt) is 0 – 0.1 kg CO2e/kg [1]. No data is available for pharmaceutical grade NaCl but according to the CarbonCloud.com lifecycle analysis 75% of the emissions associated with the manufacture of table salt are due to processing rather than extraction. To apply a realistic emissions factor we tripled the published value to account for further stages of purification to produce pharmaceutical quality NaCl and possible extra greenhouse gas (GHG) emissions from the manufacture of the Potassium, Magnesium, and Calcium chloride salts in the acid concentrate. So, we used a factor of 0.3 kg CO2e per kg NaCl for the overall salt content of the acid concentrate.

Glucose:
An emissions factor for Glucose was published in 2013 [2] and reported a range of emissions for glucose production to be between 0.7 and 1.1 kgCO2e / kg. We used the upper end of this range.

Acetic Acid:
An emissions factor for Acetic Acid of 0.61 kgCO2e / kg was published in 2015 from a life-cycle analysis [3]. The 98 litres reported by the OEM required to manufacture 10,000 litres of acid concentrate, was converted to 103kg using volume to weight converters.

Water:
According to the OEM a 10,000 litre batch of 1:44 dilution acid concentrate would contain 8,836 litres of ultrapure water. The emissions factor for potable water is 0.149 kgCO2e / m3 [4]. To account for the reject water lost during the treatment processes to produce ultrapure water and subsequent cleaning of equipment, we estimated that total losses would be 50% of the total product, as the typical reverse osmosis reject rate is around 30-35% with an extra 15-20% additional usage by water softeners and carbon filters. As such, 13,254 litres of incoming water would be required to produce each batch of concentrate.

Thus some 4418 litres of water would be rejected and discharged into the sewers for every batch produced. The appropriate emissions factor for subsequent water treatment has been included [4].

Energy:
The OEM provided invaluable data about their production process. In 2022 0.016kWh was used per litre of concentrate produced. This covered the whole process including water treatment, product mixing and the final production line. The emissions factor used for electricity is an aggregate of UK electricity generation, transmission and distribution losses and all well-to-tank factors [4].

Packaging:
Several empty 5 litre plastic containers were measured and found to weigh 250±5 g (Kern weighing scale 6000-1, Kern & Sohn GmbH, Balingen, Germany). Each container was made of non-recycled high-density polyethylene (HDPE). HDPE has an emissions factor for the formed plastic of 3.27 kgCO2e / kg [4].

Acid concentrate for a central acid delivery system (CAD) is delivered in reusable 1,000 litre containers into which is fitted a single-use liner sheet. Each liner weighing 2.275 kg is manufactured from non-recycled linear low-density polyethylene (LLDPE). For LLDPE plastic, the BEIS database provides a figure of 2.6 kgCO2e / kg for formed LLDPE [4].

The reusable container is a 1000 litre Intermediate Bulk Container (IBC) weighing approximately 58 kg and manufactured from HDPE with a steel support cage.

Transport:
An indicative figure for the emissions savings associated with the non-delivery of 40,000 litres of acid concentrate in 5-litre HDPE containers has been calculated. Assuming that all containers have 5.0 litres of acid concentrate, then 8000 containers each weighing 6.2kg would equate to 49.6 tonnes of total load. The distance from the OEM plant to the kidney care centre is almost exactly 200 km, thus there is a total of 49.6 tonnes*200km = 9920 tonne.km for the outward journey. We have assumed that the 7.5-17 tonne delivery vehicle is Average Loaded on the outward journey. So, to deliver 49.6 tonnes this will require a minimum of 3 additional journeys and therefore three return trips at zero load over 200km are included. Exhaust emissions (tail pipe) and well to tank (WTT) emissions generated from oil extraction to refinery to petrol station are included.

Waste:
The BEIS database lists an emissions factor of 21.28 kgCO2e / tonne for the disposal of all plastics, though this only covers delivery to a recycling centre [4].

**Boundaries and Omissions:**

Capital Equipment:
In line with the GHG Protocol Product Standard [5,6], no account is taken of the emissions associated with the manufacture or installation of equipment used in the production process or in the distribution equipment required for CAD. These numbers are amortised over the life of the equipment and so considered minor in the overall scheme.

Concentrate Production:
More equipment is used in the 5.0 litre plastic container production line compared to filling bulk containers, but separate energy figures are not available and are not considered large enough to make a significant impact on the overall estimates.

Transport:
No account is taken of the transport costs associated with delivering the ingredients of the concentrate or the packaging to the OEM production facility as this information is not publicly available.

For the same reason, no account is taken for the deliveries of additional chemical components and plastic packaging to the manufacturing factory for the additional 40,000 L of acid concentrate that needs to be produced if the kidney service is using individual 5 L containers of acid concentrate.

Fresenius Medical Care main deliveries are currently interwoven across their entire product range and delivery sites. They depend upon many factors such as delivery vehicle size and capacity, milage to different sites, with multiple different products on board. Due to this complexity, unfortunately, extracting any meaningful data about the overall delivery emissions is not possible.

The indicative figure for the savings associated with non-delivery of 40,000 litres will be an underestimate of the true figure. Whilst the outward journey can be calculated using the tonne.km data, only the emissions associated with three return journeys are included whereas in reality it will be more likely monthly deliveries.

Waste:
In line with GHG Protocols [5], the energy used in the recycling process is accounted for within the factors of future electricity generation.

Maintenance:
Maintenance of the production facility and of equipment associated with CAD is considered outside the scope of the study at this time.

Packaging:
An empty 5 litre HDPE container, with lid, weighs 250±5 g (Kern weighing scale 6000-1, Kern & Sohn GmbH, Balingen, Germany) and is assumed to be homogenous. No account is taken of the small difference in materials in respect of the lid and seal. No account is taken of the packaging associated with moving the containers on pallets (cardboard or clingfilm) or the labelling. These factors are considered negligible in the overall calculations.

The container used in bulk deliveries is a 1000 litre Intermediate Bulk Container (IBC) weighing approximately 58 kg and manufactured from HDPE with a steel support cage. Its construction is estimated to cause approximately 150 kgCO2e in emissions but they are used for many years and thousands of deliveries. Within this study their contribution is considered negligible.

**Results:**

We estimated the greenhouse gas emissions for the manufacture of 10,000 litres of dialysate acid concentrate was 1548 kgCO2e (Table 2). We then calculated the carbon footprint generated by the manufacture of the LLDPE plastic liners for the bulk delivery of acid concentrate (60 kgCO_2_e) and for the manufacture of individual 5 litre HDPE containers (1,646 kgCO_2_e) (Table 3). Greenhouse gas emissions for the waste disposal of plastics was based on the weight of used plastic to be delivered to a recycling centre. Table 4 outlines the carbon footprint of transporting the extra 40,000 L of acid concentrate from the factory to the dialysis centre required if using 5 L containers with the current 33% wastage (5,205 kgCO_2_e).

**Tables**

Table 1: Contents of a 10,000 litre batch of acid concentrate. Data kindly provided by OEM, Fresenius Medical Care UK Ltd.

Table 2. Estimate of the carbon footprint for the manufacture of 10,000 litres of dialysate acid concentrate: Total greenhouse gas emissions 1548 kgCO2e

| Item | Description | Unit | Carbon emission factor/unit | Carbon emission kgCO_2_e |
| --- | --- | --- | --- | --- |
| Ultra-pure water | 13254 L | m^3^ | 0.149 | 2 |
| Sodium chloride | 2924 kg | kg | 0.3 | 877 |
| Glucose | 512 kg | kg | 1.1 | 563 |
| Acetic acid | 98 L/103 kg | kg | 0.61 | 63 |
| Energy | 0.016 kWh/L | kWh/L | 0.26155 | 42 |
| Waste water | 4418 L | m^3^ | 0.272 | 1 |
| Total |  |  |  | 1548 |

Table 3. Carbon emission values estimated from the manufacture of plastic containers for 10,000 L of acid dialysate concentrate and their waste disposal.

| Item | Description | Unit | Carbon emission /item | Carbon emission kgCO_2_e |
| --- | --- | --- | --- | --- |
| Central acid delivery system | | | | |
| LLDPE | 10*1,000 L liner (2.275 kg each) | kg | 5.91 | 59 |
| Waste | 22.75 kg (10 x liners) | kg | 1.0 | 0.5 |
| Total CAD |  |  |  | 60 |
| 5.0 L single use containers | | | | |
| HDPE container | 250 g | kg | 0.8175 | 1635 |
| Waste | 400/tonne | tonne | 0.00532 | 11 |
| Total 5 L cans |  |  |  | 1646 |

Table 4. Carbon emission values estimated in delivering extra 40,000 L of acid dialysate in single use 5.0 L containers. Distance between supplier and dialysis centre 200 km.

| Item | Description | Unit | Carbon emission factor/item | Carbon emission kgCO_2_e |
| --- | --- | --- | --- | --- |
| Outward journey | | | | |
| Tailpipe | 49.6 tonne*200 km | Tonne.km | 0.38655 | 3835 |
| WTT | 49.6 tonne*200 km | Tonne.km | 0.08238 | 817 |
| Return journey | | | | |
| Tail pipe | 0 tonne 3*200 km | km | 0.79097 | 475 |
| WTT | 0 tonne 3*200 km | km | 0.12960 | 78 |
| Total difference |  |  |  | 5205 |

References:

1. Website: Carbon Cloud: [Salt (NaCl) · 0-0.1 kg CO₂e/kg | Verified by CarbonCloud](https://apps.carboncloud.com/climatehub/product-reports/id/5403842093) (Feb 2023)
2. Energy and greenhouse gas assessment of European glucose production from corn – a multiple allocation approach for a key ingredient of the bio-based economy. [Journal of Cleaner Production](https://www.researchgate.net/journal/Journal-of-Cleaner-Production-0959-6526) 43:182-190 March 2013 [10.1016/j.jclepro.2012.12.035](http://dx.doi.org/10.1016/j.jclepro.2012.12.035)
3. Adom F, Dunn JB. MATERIAL AND ENERGY FLOWS IN THE PRODUCTION OF MACRO AND MICRONUTRIENTS, BUFFERS, AND CHEMICALS USED IN BIOCHEMICAL PROCESSES FOR THE PRODUCTION OF FUELS AND CHEMICALS FROM BIOMASS Energy Systems Division Argonne National Laboratory September 30th, 2015. <https://www.google.co.uk/url?sa=t&rct=j&q=&esrc=s&source=web&cd=&ved=2ahUKEwju6dyW5Zf9AhWRolwKHRrDA50QFnoECBgQAQ&url=https%3A%2F%2Fgreet.es.anl.gov%2Ffiles%2Ffuel-chemicals-biomass&usg=AOvVaw0ssL_8861UpvpXcbo4aN29>
4. DEFRA/BEIS Database ghg-conversion-factors-2022-full-set: [Greenhouse gas reporting: conversion factors 2022 - GOV.UK (www.gov.uk)](https://www.gov.uk/government/publications/greenhouse-gas-reporting-conversion-factors-2022)
5. Greenhouse Gas Protocol. Product Standard: [Product Standard | GHG Protocol](https://ghgprotocol.org/product-standard).
6. British Standards Institute (BSI): PAS 2050-Specification for the assessment of the life cycle greenhouse gas emissions of goods and services. [PAS 2050:2011 | 30 Sep 2011 | BSI Knowledge (bsigroup.com)](https://knowledge.bsigroup.com/products/specification-for-the-assessment-of-the-life-cycle-greenhouse-gas-emissions-of-goods-and-services?version=standard)
